# Neuroprotective Effect of Clobenpropit against Lipopolysaccharide-Induced Cognitive Deficits via Attenuating Neuroinflammation and Enhancing Mitochondrial Functions in Mice

**DOI:** 10.3390/brainsci11121617

**Published:** 2021-12-08

**Authors:** Vasudevan Mani, Minhajul Arfeen, Hussein M. Ali, Abdel-Moneim Hafez Abdel-Moneim, Maha Aldubayan, Ahmad Alhowail

**Affiliations:** 1 Department of Pharmacology and Toxicology, College of Pharmacy, Qassim University, Buraydah 51452, Saudi Arabia; hu.ali@qu.edu.sa (H.M.A.); m.aldubayan@qu.edu.sa (M.A.); aalhowail@qu.edu.sa (A.A.); 2 Department of Medicinal Chemistry and Pharmacognosy, College of Pharmacy, Qassim University, Buraydah 51452, Saudi Arabia; m.arfeen@qu.edu.sa; 3 Department of Biochemistry, Faculty of Medicine, Al-Azhar University, Assiut 71524, Egypt; 4 Department of Physiology, College of Medicine, Qassim University, Buraydah 51452, Saudi Arabia; a.elmonem@qu.edu.sa; 5 Department of Physiology, Faculty of Medicine, Mansoura University, Mansoura 35516, Egypt

**Keywords:** clobenpropit, lipopolysaccharides, radial arm maze, cyclooxygenase-2, pro-inflammatory cytokines, anti-inflammatory cytokines, mitochondrial dysfunction

## Abstract

Clobenpropit (CLO), an antagonist on histamine H_3_ receptors (HH3R), has been shown to protect NMDA-induced neuronal necrosis in cortical neuronal cell culture from rats. In this work, we explored its potential on lipopolysaccharide (LPS)-induced memory deficits, neuroinflammation, and mitochondrial dysfunction in mice. CLO (1 and 3 mg/kg, p.o.) was treated continually for 30 days, and neurotoxicity was induced by four doses of LPS (250 µg/kg, i.p.). The radial arm maze (RAM) was used to access memory behaviors. After the REM test, brain tissue was collected from each mouse to estimate pro-inflammatory cytokines (TNFα and IL6), anti-inflammatory cytokines (TGF-β1 and IL-10), cyclooxygenase-2 (COX 2), and mitochondrial respiratory chain complex (MRCC- I, II and IV) enzymes. CLO treatment reversed the LPS-induced behavioral deficits by a significant reduction in time taken to consume all five bites (TTB), working memory error (WME), and reference memory error (REM) in the REM test. Regarding neuroinflammation, it attenuated the release of COX, TNF-α, and IL-6, and augmented TGF-β1 and IL-10 levels in the brain. Reversal of LPS-induced brain MRCC (I, II, and IV) levels also resulted with CLO treatment. From these findings, CLO promises neuroprotection against LPS-induced cognitive deficits by ameliorating neuroinflammation and restoring the MRCC enzymes in mice.

## 1. Introduction

Neuroinflammation is defined as one of the key contributors involved in several CNS-related disorders including neurodegenerative diseases. According to experimental evidence, the inflammatory process in the neuron has been shown to cause cell death and neurodegeneration in Parkinson’s (PD), Alzheimer’s (AD), and other neurodegenerative diseases [1,2]. Moreover, neuroinflammation and mitochondrial dysfunction also have a key role in AD and other neurodegenerative-related disorders [3,4]. In the brain, neuroinflammation is mediated by several factors, including cytokines, prostaglandin E2, oxidative stress, and reactive nitrogen species [5]. Furthermore, inflammatory mediators, particularly the cytokine TNF-α, can change cellular mitochondrial metabolism by inhibiting mitochondrial oxidative phosphorylation and related ATP synthesis while also initiating mitochondrial reactive oxygen species formation. On the other hand, when damaged mitochondria are not appropriately eliminated by mitophagy, their contents can leak into the cytosol and extracellular environment, aggravating the inflammatory responses in brain tissue [6].

Central or peripheral administration of an endotoxin, lipopolysaccharide (LPS), results in neuroinflammation by inducing the releases of cytokines including TNF-α, IL-1β, and IL-6 through activation of microglia [7]. Furthermore, the elevation of these cytokines induces the proliferation of APP expression and amyloidogenesis in AD [8]. A previous study showed that LPS stimulated the expression of neuroinflammatory markers due to differences in oxidative stress, oxidative phosphorylation, and mitochondrial activities. The mitochondrial complex activity and mitochondrial membrane potential could be influenced by oxidative stress [1]. Additionally, a bilateral intracerebroventricular injection of LPS established mitochondrial electron transfer chain dysfunction by decreasing the MRCC (I, IV, and V) activities [5].

The list of antagonists on HH3R has gained substantial attention in recent years as a potential treatment for CNS-related disorders including AD. These receptors are found in pre-synaptic neurons as auto-receptors as well as heteroreceptors and control the release of neurotransmitters including histamine [3]. Antagonizing the HH3R facilitates the pre-synaptic releases of several neurotransmitters including histamine, acetylcholine, norepinephrine, and dopamine, which have a major role in various CNS functions [9]. CLO is a potent HH3R antagonist and has been reported to augment the pre-synaptic release of neurotransmitters including histamine, acetylcholine, dopamine, and noradrenaline [10,11,12,13]. Recently, in a rat model of AD, administration of CLO (1 mg/kg, i.p.) was found to have a neuroprotective effect against Aβ-peptide infusion-induced neuronal toxicity [14]. Furthermore, in rats, a bilateral intrahippocampal injection of CLO improves spatial memory deficits caused by MK-801 via modulating the levels of different neurotransmitters [15]. However, there is evidence lacking related to the effect of CLO on neuroinflammation and mitochondrial dysfunction. Thus, the current study aimed to evaluate the effect of 30 days of CLO pre-treatment on LPS-induced cognitive deficits, neuroinflammation, and mitochondrial dysfunction in a mouse model.

## 2. Materials and Methods

### 2.1. Drugs and Chemicals

Clobenpropit hydrobromide (CLO) was purchased from Cayman Chemical (Ann Arbor, MI, USA) and lipopolysaccharides (LPS) from *Escherichia coli* were procured from Sigma-Aldrich Co (St. Louis, MO, USA). Mouse TNF-α, IL-6, TGF-β1, IL-10, and COX- 2 were purchased from Cloud-Clone Corp., Houston, TX, USA. Mouse MRCC-I, MRCC-II, and MRCC-IV were purchased from MyBioSource, Inc., San Diego, CA, USA.

### 2.2. Animals

The present experiment was used with 24 adults (8–12 weeks old) male ICR mice weighing between 25 and 35 g, procured from the Animal Facility of Pharmacology and Toxicology Department, College of Pharmacy, Qassim University, KSA. Mice were randomly dived into four groups of six animals. During the experiment, animals were housed in polypropylene cages containing three subjects per cage and had free access to water and food. The present experimental procedures were reviewed by the Institutional Animal Ethical Committee from College of Pharmacy, Qassim University, KSA (Approval ID 2020-CP-7).

### 2.3. Experimental Design

**Figure 1:**
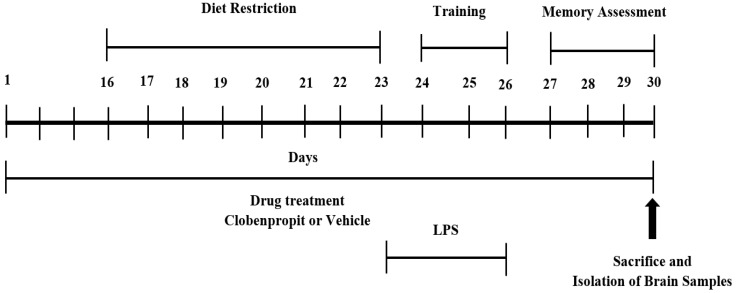
Timeline administration of drug, behavioral assessments, and isolation of brain samples. The groups of the mice were administered vehicle or clobenpropit (1 or 3 mg/kg) orally for 30 days. Except for the control, other groups were injected with four doses of LPS (250 μg/kg, i.p.) to induce neuroinflammation (days 23–26). Regarding RAM assessments, the training sessions were conducted from days 24 to 26. The memory assessments were analyzed from days 27 to 30. At the end of the memory assessments, on day 30, all the animals were sacrificed and brain tissues were collected for ELISA tests.

Among the four groups, the first group was fixed as a control and only treated with vehicle (normal saline: 10 mL/kg, p.o.) for 30 days; four doses of normal saline (10 mL/kg, i.p.) were injected for the last four days (day 23 to day 26) of the treatment schedule. The second group was LPS-treated (LPS), administered with vehicle (normal saline; 10 mL/kg, p.o.) for 30 days; neuroinflammation was induced by four doses of LPS (250 μg/kg, i.p.) on days 23, 24, 25 and 26 of treatment. The neurotoxic dose of LPS was sourced from our previous reports [16,17]. The other two treatment groups (LPS + CLO-1; LPS + CLO-3) were treated orally with CLO (1 or 3 mg/kg, respectively) for 30 days and neurotoxicity was induced with four doses of LPS (250 μg/kg, i.p.) following the LPS-treated group. During the treatment schedule, the behavioral assessments using RAM were followed in three different phases: diet restriction (day 16 to day 23), training (day 24 to day 26), and memory assessment (days 27–30), following a detailed procedure (Figure 1).

### 2.4. Behavioral Assessment Using the Radial Arm Maze (RAM)

RAM was used to measure the spatial learning and memory of mice in this study. The maze comprises eight arms (48 × 12 cm) with a connecting central platform (32 cm in diameter). The experimental procedures and parameters were followed according to earlier reports [3,16]. The procedure behavioral assessments were followed in three different phases: diet restriction, training, and memory assessment (Figure 1). Among the eight arms, 1, 3, 4, 6, and 7 were considered as baited arms, and the remaining 2, 5, and 8 were referred to as non-baited arms. Each of the arms were differentiated with dissimilar geometric-shaped paper pasted at the arm end. Since this model was food reward-based, the seven-days diet restriction (day 16 to day 23 of drug treatment) was followed to create a food motivational task. In training (day 24 to day 26), on the first day, the food pellets were dispersed throughout the maze and the animals were allowed to explore for five minutes; on subsequent days, food was allowed only in baited arms. During the last four days (day 27–30) of the treatment schedule the memory parameters like time taken to consume all five baits (TTB), working memory error (WME), and reference memory error (RME) were recorded over five minutes of exploration. The WME was counted as the total number of re-entries to baited arms that were already eaten and the total number of entries into the never-baited arm was referred to as RME [3].

### 2.5. Collection of Brain Homogenate

At the end of the 30-day treatment, all the groups of mice were sacrificed and whole-brain tissues were homogenized with ice-cold phosphate buffer (4 °C, pH 7.4) using a homogenizer. The cloudy supernatant aliquot of homogenates was collected after centrifuging for 10 min at 4000 rpm. The total protein content of the homogenates was quantified using the biuret colorimetric method (Crescent Diagnostics, Jeddah, Saudi Arabia).

### 2.6. Determination of Cytokines and Cyclooxygenase levels

Selectively, two pro-inflammatory (TNFα and IL6) and two anti-inflammatory (TGFβ1 and IL10) cytokines, and COX-2 were tested using the mouse enzyme-linked immunosorbent assay (ELISA) kit (Cloud-Clone Corp., Houston, TX, USA) protocol. The assay kits were followed by sandwich enzyme immunoassay for in vitro quantitative measurement of specifically targeted proteins.

### 2.7. Determination of Mitochondrial Respiratory Chain Complexes (MRCC)

The mouse MRCC-I, MRCC-II, and MRCC-IV ELISA kits were from MyBioSource Inc. (San Diego, CA, USA). Measurements were achieved at 450 nm by using an Absorbance Microplate Reader (ELx800, BioTek Instruments, Inc., Santa Clara, CA, USA).

### 2.8. Statistical Analysis

The results are presented here as mean ± standard error (SEM). The variations among all groups were analyzed using one-way ANOVA. We followed a Tukey–Kramer post hoc test for calculating significance levels between groups. Graph Pad version 9 (GraphPad Software Inc., La Jolla, CA, USA) was employed for statistical analysis. *p* values less than 0.05 were considered statistically significant.

## 3. Results

### 3.1. CLO Improved Memory Functions in LPS-Treated Mice Using the RAM

**Figure 2:**
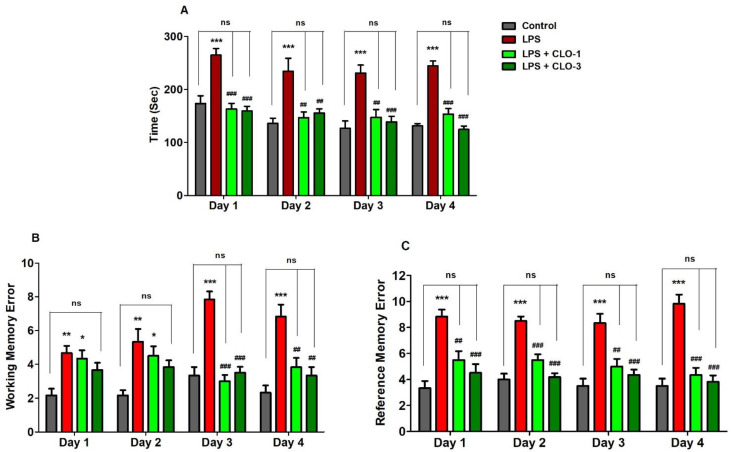
Effect of the clobenpropit (CLO) on the (**A**) time taken to consume all five baits (TTB), (**B**) working memory error (WME), and (**C**) reference memory error (RME) on day 1 to day 4 of memory assessment in lipopolysaccharide (LPS)-induced mice using radial arm maze. LPS-CLO-1 and LPS-CLO-3 refer to administration of clobenpropit (1 or 3 mg/kg, p.o., respectively) and lipopolysaccharides (250 μg/kg, i.p.). TTB, WME and REM were increased by LPS-induced neuroinflammation. However, CLO treatment significantly reduced the LPS-induced TTB, WME and REM increments. The results are expressed as mean ± SEM (*n* = 6). One-way ANOVA (TTB; *F*(3,20) = 17.87, *p* < 0.001 for day 1; *F*(3,20) = 9.030, *p* < 0.001 for day 2; *F*(3,20) = 11.74, *p* < 0.001 for day 3; and *F*(3,20) = 25.95, *p* < 0.001 for day 4), (WME; *F*(3,20) = 6.460, *p* < 0.01 for day 1; *F*(3,20) = 6.272, *p* < 0.01 for day 2; *F*(3,20) = 28.97, *p* < 0.001 for day 3; and *F*(3,20) = 12.39, *p* < 0.001 for day 4) (REM; *F*(3,20) = 14.88, *p* < 0.001 for day 1; *F*(3,20) = 29.21, *p* < 0.001 for day 2; *F*(3,20) = 13.41, *p* < 0.001 for day 3; and *F*(3,20) = 26.52, *p* < 0.001 for day 4) was followed by Tukey–Kramer multiple comparisons tests.* *p* < 0.05, ** *p* < 0.01, and *** *p* < 0.001 as compared to the control group; ns, not significant as compared to the control group; ^##^ *p* < 0.01 and ^###^ *p* < 0.001 as compared to the LPS-treated group.

Memory functions of LPS-challenged mice pre-treated with CLO were examined in terms of selected three behavioral parameters such as TTB, WME, and RME in the RAM test. Figure 2A shows the effect of CLO on four days of TTB assessment in LPS-induced memory impairment. Analyzing by one-way ANOVA and comparing among the groups, statistical differences were found in TTB (*F*(3,20) = 17.87, *p* < 0.001 for day 1; *F*(3,20) = 9.030, *p* < 0.001 for day 2; *F*(3,20) = 11.74, *p* < 0.001 for day 3; and *F*(3,20) = 25.95, *p* < 0.001 for day 4). Furthermore, multiple post hoc analyses indicated that LPS-treatment extensively increased (*p* < 0.001) the TTB for four days as compared with the control, representing the memory decline by LPS injections. However, CLO at doses of 1 mg/kg (*p* < 0.001, day 1 and day 4; *p* < 0.01, day 2 and day 3) and 3 mg/kg (*p* < 0.001, day 1, day 3, and day 4; *p* < 0.01, day 2) significantly reduced the LPS-induced TTB increase.

As shown in Figure 2B, when comparing among groups, significant variations were noted in the number of WME from day 1 to day 4 (*F*(3,20) = 6.460, *p* < 0.01; *F*(3,20) = 6.272, *p* < 0.01; *F*(3,20) = 28.97, *p* < 0.001; *F*(3,20) = 12.39, *p* < 0.001, respectively). Administration of LPS by peripheral injections impaired the working memory by increasing the WME (*p* < 0.01, day 1 and day 2; *p* < 0.001, day 3 and day 4) throughout the experiment. Pre-administration of CLO, however, considerably reduced the number of WME only on day 3 and day 4 (*p* < 0.001 and *p* < 0.01 at 1 and 3 mg/kg, p.o., respectively).

In addition, the reference memory was also altered with different groups of treatments (*F*(3,20) = 14.88, *p* < 0.001 for day 1; *F*(3,20) = 29.21, *p* < 0.001 for day 2; *F*(3,20) = 13.41, *p* < 0.001 for day 3; and *F*(3,20) = 26.52, *p* < 0.001 for day 4) by using one-way ANOVA analysis (Figure 2C). When matched to control animals, it was indicated that the LPS-treated mice group showed higher numbers of RMEs (*p* < 0.001) after four days of the experiments. Nevertheless, additional treatment of CLO with the LPS treatment extensively reduced the number of RMEs at the oral dose levels of 1 mg/kg (*p* < 0.01, day 1 and day 3; *p* < 0.001, day 2 and day 4) and 3 mg/kg (*p* < 0.001, from day 1 to day 4).

### 3.2. CLO Reduced Pro-Inflammatory Cytokine Levels in LPS-Treated Mice

**Figure 3:**
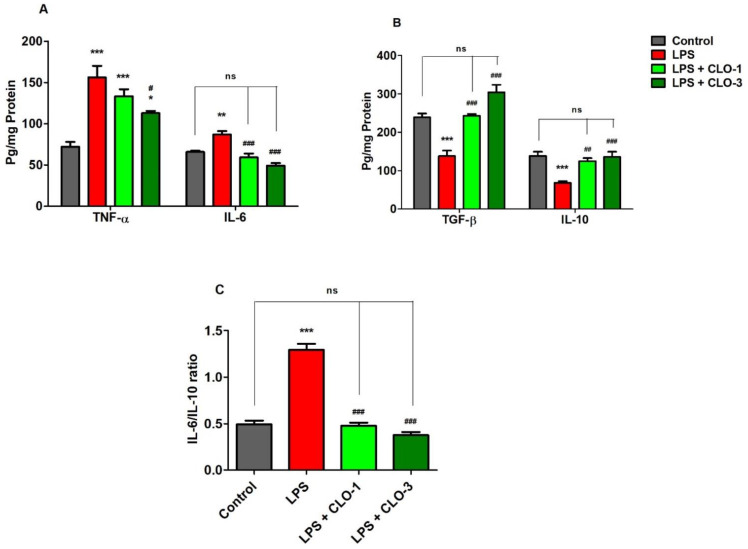
Effect of clobenpropit (CLO) on (**A**) pro-inflammatory cytokines (TNF-α and IL-6), (**B**) anti-inflammatory cytokines (TGF-β and IL-10), and (**C**) IL-6/IL-10 ratio in lipopolysaccharide (LPS)-induced mice brains. LPS-CLO-1 and LPS-CLO-3 refer to the administration of clobenpropit (1 or 3 mg/kg, p.o, respectively) and lipopolysaccharides (250 μg/kg, i.p.). Administration of LPS resulted in the elevation of pro-inflammatory cytokines and reduction of anti-inflammatory cytokine activities. Interestingly, the CLO treatment attenuated the inflammatory activity of LPS. The ratio of IL-6/IL-10 also lowered with CLO treatment in LPS-challenged mice. The results are expressed as mean ± SEM (*n* = 6). One-way ANOVA (*F*(3,20) = 16.68, *p* < 0.001 for TNF-α and *F*(3,20) = 20.96, *p* < 0.001 for IL-6) (*F*(3,20) = 26.33, *p* < 0.001 for TGF-β and *F*(3,20) = 11.18, *p* < 0.001 for IL-10) (*F*(3,20) = 88.18, *p* < 0.001 for IL-6/IL-10 ratio) was followed by Tukey–Kramer multiple comparisons tests. * *p* < 0.05, ** *p* < 0.01 and *** *p* < 0.001 as compared to the control group; ns, not significant as compared to the control group; ^#^ *p* < 0.05, ^##^ *p* < 0.01 and ^###^ *p* < 0.001 as compared to the LPS-treated group.

Figure 3A demonstrates the effects of CLO on two selective pro-inflammatory cytokine markers, TNF-α and IL-6. Comparing all the groups, considerable differences were found in TNF-α levels (*F*(3,20) = 16.68, *p* < 0.001) by analyzing with one-way ANOVA. Compared to the control, the LPS-challenged group elicited significantly higher (*p* < 0.001) TNF-α levels in the brain. CLO treatment (3 mg/kg, p.o.) considerably reduced (*p* < 0.05) the production of TNF-α in mice brains, as compared to the LPS-challenged group. There were no obvious changes in TNF-α levels with administration of CLO (1 mg/kg, p.o.) in LPS-challenged mice.

The effect of CLO on brain IL-6 production in LPS-treated mice is shown in Figure 3A. Referring to the results, when comparing among groups, differences in IL-6 levels (*F*(3,20) = 20.96, *p* < 0.001) were found in brain homogenates. Multiple post hoc comparisons showed that LPS administration significantly elevated (*p* < 0.01) IL-6 levels in brain tissues of the control animals. However, treatment with CLO (1 and 3 mg/kg, p.o.) suggestively (*p* < 0.001) reversed it by reducing the brain IL-6 levels when compared with the LPS-treated group, and both doses almost equalized the IL-6 levels of the control. 

### 3.3. CLO Improved Anti-Inflammatory Cytokine Levels in LPS-Treated Mice

Figure 3B shows the effects of CLO on anti-inflammatory cytokine markers TGF-β1 and IL-10 in brain homogenates of LPS-treated mice. There were extensive differences noted when comparing among the groups of TGF-β1 levels (*F*(3,20) = 26.33, *p* < 0.001). The levels of TGF-β1 in LPS-injected mice were significantly (*p* < 0.001) lower compared with control mice. The results indicated that LPS-treatment lowered the anti-inflammatory activity in the mouse brain. Pretreatment with 1 and 3 mg/kg of CLO, however, significantly improved TGF-β1 levels in LPS-challenged mice (*p* < 0.01 and *p* < 0.001, respectively). There were no notable changes between the control and both CLO-treated groups.

For IL-10 levels, there were significant variations recorded (*F*(3,20) = 11.18, *p* < 0.001) among the groups (Figure 3B). When compared with the control group, it was shown that LPS treatment resulted in a significantly lower level of IL-10 (*p* < 0.001) in mice brains. Nevertheless, CLO (1 and 3 mg/kg, p.o.) pre-treatment substantially elucidated *(p* < 0.01 and *p* < 0.001) the brain IL-10 levels in LPS-challenged animals.

Furthermore, the effects of CLO on the IL-6/IL-10 ratio in LPS-treated mice are shown in Figure 3C. The IL-6/IL-10 ratio was significantly (*p* < 0.001) higher in LPS-treated mice than in the control. This indicated the higher activity of pro-inflammatory cytokines with LPS treatment. However, treatment with 1 and 3 mg/kg of CLO significantly (*p* < 0.001) lowered the IL-6/IL-10 ratio in LPS-challenged mice.

### 3.4. CLO Reduced Cyclooxygenase-2 (COX-2) Activities in LPS-Treated Mice

**Figure 4:**
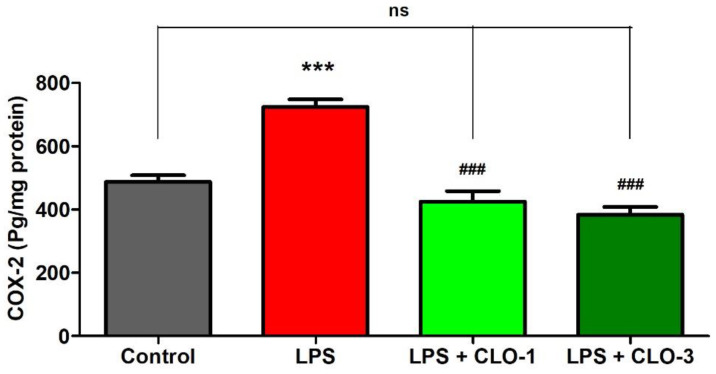
Effect of clobenpropit (CLO) on cyclooxygenase-2 (COX-2) levels in lipopolysaccharide (LPS)-induced mice brains. LPS-CLO-1 and LPS-CLO-3 refer to administration of clobenpropit (1 or 3 mg/kg, p.o., respectively) and lipopolysaccharides (250 μg/kg, i.p.). CLO treatment reduced the activity of COX-2 induced by LPS. The results are expressed as mean ± SEM (*n* = 6). One-way ANOVA (*F*(3,20) = 33.67, *p* < 0.001) was followed by Tukey–Kramer multiple comparisons tests. *** *p* < 0.001 as compared to the control group; ns, not significant as compared to the control group; ^###^ *p* < 0.001 as compared to the LPS-treated group.

Figure 4 refers to the effects of CLO on brain COX-2 levels of LPS-treated mice. While comparing among groups, extensive differences were shown for COX-2 levels (*F*(3,20) = 33.67, *p* < 0.001) using one-way ANOVA analysis. Furthermore, the comparison between selected groups showed a significant raise (*p* < 0.001) in COX-2 enzyme levels of LPS-challenged mice brains compared to the control group. However, oral treatment with CLO considerably reduced (*p* < 0.001) COX-2 activity in LPS-challenged mice. In both CLO treatment groups, the COX-2 levels were similar to the control animals.

### 3.5. CLO Improved Mitochondrial Respiratory Chain Complexes (MRCC) Activities in LPS-Treated Mice

**Figure 5:**
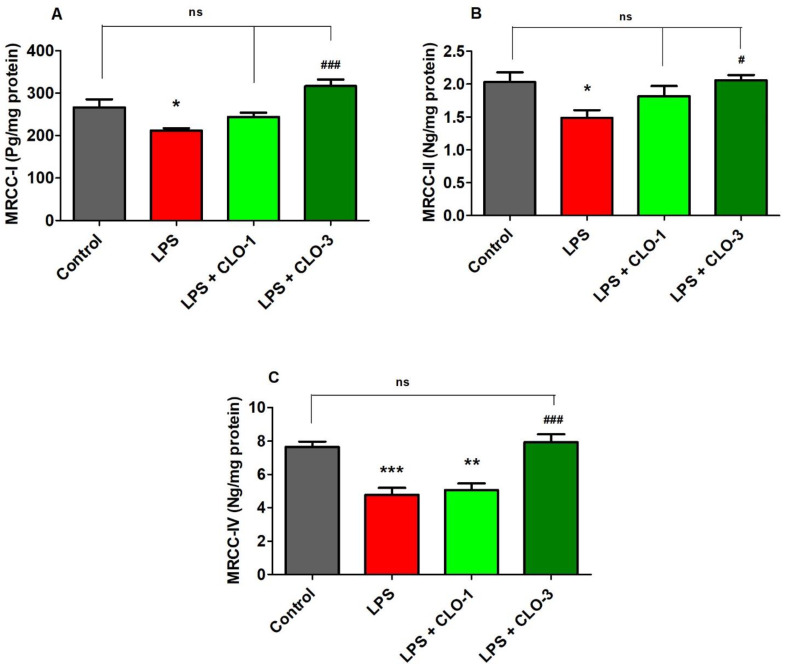
Effect of clobenpropit (CLO) on mitochondrial respiratory chain complex (MRCC) (**A**) MRCC-I, (**B**) MRCC-II, and (**C**) MRCC-IV activities in lipopolysaccharides (LPS)-treated mice brains. LPS-CLO-1 and LPS-CLO-3 refer to the administration of clobenpropit (1 or 3 mg/kg, p.o., respectively) and lipopolysaccharides (250 μg/kg, i.p.). MRCC levels were decreased with administration of LPS injection and the levels were increased with oral administration of CLO. The results are expressed as mean ± SEM (*n* = 6). One-way ANOVA (*F*(3,20) = 10.76, *p* < 0.001 for MRCC-I; *F*(3,20) = 4.270, *p* < 0.05 for MRCC-II; *F*(3,20) = 16.85, *p* < 0.001 for MRCC-IV) was followed by Tukey–Kramer multiple comparisons tests. * *p* < 0.05, ** *p* < 0.01 and *** *p* < 0.001 as compared to the control group; ns, not significant as compared to the control group; ^#^ *p* < 0.05 and ^###^ *p* <0.001 as compared to the LPS-treated group.

Figure 5 shows the effect of LPS and CLO treatment on various MRCC activities. Regarding MRCC-I, one-way ANOVA analysis showed that there was considerable variation (*F*(3,20) = 10.76, *p* < 0.001) among the groups (Figure 5A). Post hoc analysis revealed that i.p. injection of LPS caused a significant decrease (*p* < 0.01) in MRCC-I activity in mice brains. Nevertheless, treatment with CLO at 3 mg/kg (*p* < 0.001) attenuated the LPS-induced declined MRCC-I activity in the brain. There were no changes in brain MRCC-I activity when compared with LPS-treatment.

Results from MRCC-II activity (Figure 5B), when compared among the groups, show that there were significant changes in MRCC-II activity (*F*(3,20) = 4.270, *p* < 0.05) among the groups in mice brains. Furthermore, administration of LPS caused a considerable decrease in MRCC-II activity as related to control animals. Similarly, the higher dose of CLO (3 mg/kg, p.o.) significantly improved the brain MRCC-II activity (*p* < 0.05) that was decreased by injections of LPS. At a low dose of CLO (1 mg/kg, p.o.), no alteration in the level of brain MRCC-II activity resulted as compared to LPS-treated mice.

It was found (Figure 5C) that there were notable variations among the groups for brain MRCC-IV activity (*F*(3,20) = 16.85, *p* < 0.001) in mice. When compared to the control group, the LPS-treatment declined the MRCC-IV activity (*p* < 0.001) in brain tissues. Interestingly, treatment with both doses (1 and 3 mg/kg, p.o.) of CLO reversed the MRCC-IV levels (*p* < 0.01 and *p* < 0.001, respectively) in a dose-dependent manner in mice brains.

## 4. Discussion

In this present study, we evidenced the possible effects of an HH3R antagonist clobenpropit (CLO) on LPS-induced neuronal toxicity such as memory deficits, neuroinflammation, and mitochondrial dysfunction in mice. Most of our results supported the neuroprotective effects of CLO by ameliorating memory impairment, neuroinflammation, and mitochondrial toxicity, shown by reducing cyclooxygenase 2 (COX-2) and pro-inflammatory cytokines. On the other hand, CLO treatment improved memory functions, anti-inflammatory cytokine, and mitochondrial respiratory chain complex (MRCC) activities in LPS-challenged mouse brain. As described in our recent reports, peripheral injection of LPS induced memory deficits through triggering neuroinflammation by regulating COX activities as well as cytokine levels in the brain [17,18]. Moreover, brain mitochondrial functions were also altered by the i.p. administration of LPS in rodent models [19,20]. Parallel to these results, our findings confirmed the induction of neuroinflammation by elevating COX-2, TNF-α, and IL-6 synthesis, while reducing TGF-β1 and IL-10 levels in the mouse brain. Additionally, MRCC (I, II, and IV) activities also declined, accompanied by LPS toxicities. Since systemic chronic inflammation is a high-risk factor for neuronal damage and memory impairment that leads to neurodegenerative disorders including AD and PD, it appears that paths to alleviating inflammation have some benefits. The recent finding has shown that HH3R is highly found not only in the neurons, but also in astrocytes and microglia. Moreover, members of HH3R antagonists have shown their anti-dementia and anti-inflammatory efficacy in various experimental models [3,21].

Here, a RAM test was used to assess mouse spatial learning and memory. This maze model mainly supports the assessment of two kinds of memories, namely, reference memory and working memory [22]. LPS is a toxic product from Gram-negative bacteria that can induce a range of cognitive deficits in various maze models by inducing neurotoxicity including cholinergic dysfunction, neuronal inflammation, and oxidative stress [17,18]. Recently, the activation of microglia by LPS has been related to neuronal cell loss in mouse hippocampus and memory impairment [23]. Parallel to our previous reports [17,18], the present finding also supported the induction of spatial memory impairment by peripheral injection of LPS alone resulting in a reduction of TTB, WME, and RME on four days of the experiments. In addition, the groups of mice treated with both doses of CLO (1 and 3 mg/kg, p.o.) improved all the parameters such as TTB, WME, and RME compared to the LPS-treated mice. These results suggest that the animals in the CLO-treated groups took less time to ingest all baits and avoided memory errors, implying that they were able to recall the cues and recognize the maze. WME evaluates the ability of the mouse to recall the position of arms that have already been visited in a session. As a result, the working memory is also known as short-term memory for an object, place, or stimulus that is employed within a testing session where the information to be recalled varies with each trial [3,24]. In addition, the capability of the mouse to remember the position of baited arms is used to assess REM, and also reference memory is classified as long-term memory because it is learned through repeated training and the knowledge remains consistent between trials [3,24]. Hence, the enhancement of short- and long-term memory was confirmed by a substantial decrease in both WME and RME in LPS-treated mice following CLO administration using a mouse model. The significant changes in both WME and RME were noticed only on day 3 and day 4 of memory assessments. In continuation, the present study extended this by exploring the possible mechanisms for CLO-induced anti-dementia potential against LPS-induced neurotoxicity.

Administration of LPS can induce a collection of cellular damage due to dysregulation of immune-inflammatory responses to an infective stimulus [1]. The generation of pro-inflammatory mediators and cytokines such as iNOS, COX-2, IL-1, IL-2, IL-6, IFN-γ, and TNF-α has been documented after a single or multiple systemic doses of LPS [24]. Furthermore, the cytokines such as TNF-α and IL-6, which are generated as a result of the systemic inflammatory response, which was induced here by peripheral LPS injection, can enter the CNS via the bloodstream [25]. Furthermore, the COX enzymes and the subsequent synthesis of bioactive prostaglandins from arachidonic acid undoubtedly play a role in neuroinflammation. COX-2 is an inducible enzyme that is activated by inflammatory stimuli such as cytokines and mitogens [3,26]. In microglia, the expression of COX-2 levels was mediated by the activation of cytokines such as IL-1β, TNF-α, and IL-6 [27]. Moreover, inhibition of COX-2 by a specific COX-2 inhibitor (NS-398) resulted in the suppression of upregulated IL-6 and TNF-α levels in LPS-challenged BV-2 microglial cells [28]. Our previous studies also have suggested that the administration of LPS triggered the production of COX-2, TNF-α, and IL-6 levels in brain tissues [17,18]. The current results demonstrated that treatment with CLO at 3 mg/kg significantly reduced the brain TNF-α levels in LPS-challenged mice. Moreover, attenuation of LPS-induced brain COX-2 and IL-6 levels resulted in the treatments of both dose levels (1 and 3 mg/kg, p.o.).

In reference to our previous study, the four consecutive i.p. injections of LPS (250 µg/kg) suppressed the activity of anti-inflammatory cytokine markers such as TGF-β1 and IL-10 in rodents’ brains [17,18]. Similar to these results, the other studies also have supported diminishing cytokines IL-10 and TGF-β1 levels by LPS treatment [29,30]. The cytokine, TGF-β1 is a key regulator of cell proliferation, differentiation, and extracellular matrix formation. TGF-β1 also inhibits the proliferation and differentiation of T and B cells, as well as the generation of IL-2, TNF, and IFN- γ [31]. Another cytokine, IL-10, produces anti-inflammatory responses by limiting the synthesis of proinflammatory cytokines including IL-1 and TNF-α, decreasing cytokine receptor expression, and blocking receptor activation in the brain. Furthermore, IL-10 suppresses the generation of proinflammatory cytokines by Aβ as well as LPS activities [32]. Our current results showed that LPS administration was linked with decreasing TGF-β1 and IL-10 levels in the mouse brain, and CLO (1 and 3 mg/kg, p.o.) treatment significantly improved both cytokine levels in the brain, supporting its anti-inflammatory effects.

Mitochondria are the eukaryotic cell’s power stations, producing ATP through oxidative phosphorylation from the energy released by glucose and other carbohydrates’ oxidation. The mitochondrial respiratory chain comprises five enzymatic complexes (I–V) [33]. Complex I, also known as NADH-ubiquinone oxidoreductase, is involved in the initial step of the oxidative phosphorylation pathway. Furthermore, with normal aging and neurodegenerative diseases, its activity declines considerably [34]. Succinate dehydrogenase (SDH), as a part of the respiratory chain’s complex II, intersects the tricarboxylic acid cycle and mitochondrial oxidative phosphorylation [35]. Cytochrome c-oxidase refers to complex IV, which is considered the final enzyme of the mitochondrial respiratory chain. Its function is correlated with the regulation of aerobic energy production [36]. Furthermore, mitochondria can play a role in inflammatory responses in several ways. A significant increase in cellular energy demand in the immune response is largely met by mitochondria. Recent study results demonstrated that inhibition of mitochondrial complexes, specifically complex IV, potentiated LPS-induced IL-6 levels and altered the IL-6/TNF-α ratio in human blood leukocytes [37]. The other results supported that the pro/anti-inflammatory cytokine ratios, notably the IL-1/IL-10 as well as IL-6/IL-10 ratios, were dramatically raised by inhibition of complex IV, indicating an overactive inflammatory response. The present results also showed that increasing the IL-6/IL-10 ratio in the LPS-challenged mouse brain might coincide with mitochondrial dysfunction. The significant reduction of the IL-6/IL-10 ratio by CLO treatment might influence the improvement of mitochondrial functions. Furthermore, inhibiting complex I increased both IL-1 and IFN-γ levels, showing that pro-inflammatory cytokines may be regulated by a similar mitochondrial signaling mechanism [37,38]. Continuously, a single dosage of LPS injection (250 µg/mouse, i.p.) caused an acute systemic inflammation in the mouse brain, which resulted in mitochondrial damage in the form of a substantial decrease in membrane potential and loss of mitochondrial redox function [39]. Similarly, in our results, the LPS treatment significantly reduced the MRCC (I, II, and IV) levels in the mice brains. Interestingly, treatment with a higher dose of CLO (3 mg/kg, p.o.) significantly reversed the mitochondrial damage by improving the MRCC levels in LPS-challenged mice. Accordingly, these results suggest the region-specific potential of COL in the LPS-treated neuroinflammatory mouse model.

The present study has some limitations; most of the biochemical parameters were analyzed using whole-brain samples, not focused on a specific area such as the hippocampus, cortex, etc. This work is an initial evaluation to explore the neuroprotective potential of CLO on neuroinflammation and mitochondrial dysfunction. Moreover, cognitive dysfunction is not only related to a specific area of the brain, but deficits of other areas are also interlinked with memory functions. Additionally, inflammatory insult affects the entire area of the brain. However, the current results support the further evaluation of CLO on more specific targets including memory functions.

## 5. Conclusions

Overall, our results demonstrated that using a mouse model, the HH3R antagonist clobenpropit could act as a promising neuroprotective target against cognitive impairment, neuronal inflammation, and mitochondrial damage in the brain. Clobenpropit showed improvement in spatial learning and memory in the RAM test. It also showed anti-inflammatory potential by attenuating LPS-induced elevation of COX-2 enzymes as well as pro-inflammatory cytokine levels (TNF-α, and IL-6) levels and also increased anti-inflammatory cytokine levels (TGF-β1 and IL-10) in the mouse brain. Furthermore, pre-treatment with clobenpropit (3 mg/kg, p.o.) improved the MRCC (I, II, and IV) functions in LPS-challenged mice. The achieved results underline that clobenpropit could be a promising drug in the prevention of neuroinflammatory insults in various neurodegenerative diseases.

## Data Availability

The data presented in this study are available from the corresponding author upon reasonable request.

## References

[1] Tripathi A., Paliwal P., Krishnamurthy S. (2017). Piracetam Attenuates LPS-Induced Neuroinflammation and Cognitive Impairment in Rats. Cell Mol. Neurobiol..

[2] Kwon H.S., Koh S.-H. (2020). Neuroinflammation in neurodegenerative disorders: The roles of microglia and astrocytes. Transl. Neurodegener..

[3] Mani V., Jaafar S.M., Azahan N.S.M., Ramasamy K., Lim S.M., Ming L.C., Majeed A.B.A. (2017). Ciproxifan improves cholinergic transmission, attenuates neuroinflammation and oxidative stress but does not reduce amyloid level in transgenic mice. Life Sci..

[4] Wang Y., Xu E., Musich P., Lin F. (2019). Mitochondrial dysfunction in neurodegenerative diseases and the potential countermeasure. CNS Neurosci. Ther..

[5] Joshi R., Garabadu D., Teja G.R., Krishnamurthy S. (2014). Silibinin ameliorates LPS-induced memory deficits in experimental animals. Neurobiol. Learn. Mem..

[6] van Horssen J., van Schaik P., Witte M. (2019). Inflammation and mitochondrial dysfunction: A vicious circle in neurodegenerative disorders?. Neurosci. Lett..

[7] Marefati N., Beheshti F., Memarpour S., Bayat R., Shafei M.N., Sadeghnia H.R., Ghazavi H., Hosseini M. (2020). The effects of acetyl-11-keto-β-boswellic acid on brain cytokines and memory impairment induced by lipopolysaccharide in rats. Cytokine.

[8] Ko C.-Y., Chang L.-H., Lee Y.-C., Sterneck E., Cheng C.-P., Chen S.-H., Huang A.-M., Tseng J.T., Wang J.-M. (2010). CCAAT/enhancer binding protein delta (CEBPD) elevating PTX3 expression inhibits macrophage-mediated phagocytosis of dying neuron cells. Neurobiol. Aging.

[9] Gemkow M.J., Davenport A.J., Harich S., Ellenbroek B., Cesura A., Hallett D. (2009). The histamine H3 receptor as a therapeutic drug target for CNS disorders. Drug Discov. Today.

[10] Jansen F.P., Mochizuki T., Yamamoto Y., Timmerman H., Yamatodani A. (1998). In vivo modulation of rat hypothalamic histamine release by the histamine H3 receptor ligands, immepip and clobenpropit. Effects of intrahypothalamic and peripheral application. Eur. J. Pharmacol..

[11] Munzar P., Tanda G., Justinova Z., Goldberg S.R. (2004). Histamine H3 Receptor Antagonists Potentiate Methamphetamine Self-Administration and Methamphetamine-Induced Accumbal Dopamine Release. Neuropsychopharmacology.

[12] Blandina P., Giorgetti M., Bartolini L., Cecchi M., Timmerman H., Leurs R., Pepeu G., Giovannini M. (1996). Inhibition of cortical acetylcholine release and cognitive performance by histamine H3 receptor activation in rats. Br. J. Pharmacol..

[13] Aquino-Miranda G., Osorio-Espinoza A., Escamilla-Sánchez J., González-Pantoja R., Ortiz J., Arias-Montaño J.-A. (2012). Histamine H3 receptors modulate depolarization-evoked [3H]-noradrenaline release from rat olfactory bulb slices. Neuropharmacology.

[14] Patnaik R., Sharma A., Skaper S.D., Muresanu D.F., Lafuente J.V., Castellani R.J., Nozari A., Sharma H.S. (2017). Histamine H3 Inverse Agonist BF 2649 or Antagonist with Partial H4 Agonist Activity Clobenpropit Reduces Amyloid Beta Peptide-Induced Brain Pathology in Alzheimer’s Disease. Mol. Neurobiol..

[15] Huang Y.-W., Hu W.-W., Chen Z., Zhang L.-S., Shen H.-Q., Timmerman H., Leurs R., Yanai K. (2004). Effect of the histamine H3-antagonist clobenpropit on spatial memory deficits induced by MK-801 as evaluated by radial maze in Sprague–Dawley rats. Behav. Brain Res..

[16] Delcourt J., Miller N.Y., Couzin I.D., Garnier S. (2018). Methods for the effective study of collective behavior in a radial arm maze. Behav. Res. Methods.

[17] Rahim N.S., Lim S.M., Mani V., Hazalin N.A.M.N., Majeed A.B.A., Ramasamy K. (2020). Virgin Coconut Oil-Induced Neuroprotection in Lipopolysaccharide-Challenged Rats is Mediated, in Part, Through Cholinergic, Anti-Oxidative and Anti-Inflammatory Pathways. J. Diet. Suppl..

[18] Mohd Azahan N.S., Mani V., Ramasamy K., Lim S.M., Johari James R.M., Alsharidah M., Alhowail A., Abdul Majeed A.B. (2020). Mahanimbine-induced neuroprotection via cholinergic system and at-tenuated amyloidogenesis as well as neuroinflammation in lipopolysaccharides-induced mice. Pharmacog. Mag..

[19] Bullón P., Román-Malo L., Marín-Aguilar F., Alvarez-Suarez J.M., Giampieri F., Battino M., Cordero M.D. (2014). Lipophilic antioxidants prevent lipopolysaccharide-induced mitochondrial dysfunction through mitochondrial biogenesis improvement. Pharmacol. Res..

[20] Abdel-Salam O.M.E., Abdel-Rahman R.F., Sleem A., Farrag A.R. (2011). Modulation of lipopolysaccharide-induced oxidative stress by capsaicin. Inflammopharmacology.

[21] Wang J., Liu B., Xu Y., Luan H., Wang C., Yang M., Zhao R., Song M., Liu J., Sun L. (2022). Thioperamide attenuates neuroinflammation and cognitive impairments in Alzheimer’s disease via inhibiting gliosis. Exp. Neurol..

[22] Mei J., Kohler J., Winter Y., Spies C., Endres M., Banneke S., Emmrich J.V. (2019). Automated radial 8-arm maze: A voluntary and stress-free behavior test to assess spatial learning and memory in mice. Behav. Brain Res..

[23] Kongsui R., Sriraksa N., Thongrong S. (2020). The Neuroprotective Effect of Zingiber cassumunar Roxb. Extract on LPS-Induced Neuronal Cell Loss and Astroglial Activation within the Hippocampus. BioMed Res. Int..

[24] Rubio-Perez J.M., Morillas-Ruiz J.M. (2012). A Review: Inflammatory Process in Alzheimer’s Disease, Role of Cytokines. Sci. World J..

[25] Perry V. (2004). The influence of systemic inflammation on inflammation in the brain: Implications for chronic neurodegenerative disease. Brain Behav. Immun..

[26] O’Banion M.K. (1999). Cyclooxygenase-2: Molecular Biology, Pharmacology, and Neurobiology. Crit. Rev. Neurobiol..

[27] Consilvio C., Vincent A.M., Feldman E.L. (2004). Neuroinflammation, COX-2, and ALS—A dual role?. Exp. Neurol..

[28] Zhu J., Li S., Zhang Y., Ding G., Zhu C., Huang S., Zhang A., Jia Z., Li M. (2018). COX-2 contributes to LPS-induced Stat3 activation and IL-6 production in microglial cells. Am. J. Transl. Res..

[29] Zhao D., Zhang L.J., Huang T.Q., Kim J., Gu M.-Y., Yang H.O. (2021). Narciclasine inhibits LPS-induced neuroinflammation by modulating the Akt/IKK/NF-κB and JNK signaling pathways. Phytomedicine.

[30] Brochu M.-E., Girard S., Lavoie K., Sébire G. (2011). Developmental regulation of the neuroinflammatory responses to LPS and/or hypoxia-ischemia between preterm and term neonates: An experimental study. J. Neuroinflammation.

[31] Dudchenko P.A. (2004). An overview of the tasks used to test working memory in rodents. Neurosci. Biobehav. Rev..

[32] Szczepanik A.M., Funes S., Petko W., Ringheim G.E. (2001). IL-4, IL-10 and IL-13 modulate A beta(1--42)-induced cytokine and chemokine production in primary murine microglia and a human monocyte cell line. J. Neuroimmunol..

[33] Vercellino I., Sazanov L.A. (2021). The assembly, regulation and function of the mitochondrial respiratory chain. Nat. Rev. Mol. Cell Biol..

[34] Pollard A.K., Craig E.L., Chakrabarti L. (2016). Mitochondrial Complex 1 Activity Measured by Spectrophotometry Is Reduced across All Brain Regions in Ageing and More Specifically in Neurodegeneration. PLoS ONE.

[35] Hoekstra A.S., Bayley J.-P. (2013). The role of complex II in disease. Biochim. Biophys. Acta (BBA) Bioenerg..

[36] Fontanesi F., Soto I.C., Horn D., Barrientos A. (2006). Assembly of mitochondrial cytochromec-oxidase, a complicated and highly regulated cellular process. Am. J. Physiol. Physiol..

[37] Karan K.R., Trumpff C., McGill M.A., Thomas J.E., Sturm G., Lauriola V., Sloan R.P., Rohleder N., Kaufman B.A., Marsland A.L. (2020). Mitochondrial respiratory capacity modulates LPS-induced inflammatory signatures in human blood. Brain Behav. Immun. Health.

[38] Dumitru C., Kabat A.M., Maloy K.J. (2018). Metabolic Adaptations of CD4+ T Cells in Inflammatory Disease. Front. Immunol..

[39] Noble F., Rubira E., Boulanouar M., Palmier B., Plotkine M., Warnet J.M., Marchand-Leroux C., Massicot F. (2007). Acute systemic inflammation induces central mitochondrial damage and mnesic deficit in adult Swiss mice. Neurosci. Lett..

